# High Perceived Stress May Shorten Activated Partial Thromboplastin Time and Lead to Worse Clinical Outcomes in Patients With Coronary Heart Disease

**DOI:** 10.3389/fcvm.2021.769857

**Published:** 2021-11-29

**Authors:** Han Yin, Xingyu Cheng, Yanting Liang, Anbang Liu, Haochen Wang, Fengyao Liu, Lan Guo, Huan Ma, Qingshan Geng

**Affiliations:** ^1^Department of Cardiology, Guangdong Cardiovascular Institute, Guangdong Provincial People's Hospital, Guangdong Academy of Medical Sciences, Guangzhou, China; ^2^School of Medicine, South China University of Technology, Guangzhou, China; ^3^Department of Cardiac Rehabilitation, Guangdong Cardiovascular Institute, Guangdong Provincial People's Hospital, Guangdong Academy of Medical Sciences, Guangzhou, China

**Keywords:** perceived stress, coronary heart disease, activated partial thromboplastin time, acute coronary syndrome, depression

## Abstract

**Objective:** To determine the association of perceived stress with coagulation function and their predictive values for clinical outcomes.

**Methods:** This prospective cohort study derived from a cross-sectional study for investigating the psychological status of inpatients with suspicious coronary heart disease (CHD). In this study, the 10-item Perceived Stress Scale (PSS-10) as an optional questionnaire was used to assess the severity of perceived stress. Coagulation function tests, such as activated partial thromboplastin time (APTT), prothrombin time (PT), and fibrinogen were measured within 1 h after admission. Furthermore, 241 patients with CHD out of 705 consecutive inpatients were included in the analyses and followed with a median of 26 months for the clinical outcomes.

**Results:** The patients in high perceived stress status (PSS-10 score > 16) were with shorter APTT (36.71 vs. 38.45 s, *p* = 0.009). Shortened APTT ( ≤ 35.0 s) correlated with higher PSS-10 score (14.67 vs. 11.22, *p* = 0.003). The association of APTT with depression or anxiety was not found. Multiple linear models adjusting for PT estimated that every single point increase in PSS-10 was relevant to approximately 0.13 s decrease in APTT (*p* = 0.001) regardless of the type of CHD. APTT (every 5 s increase: hazard ratio (*HR*) 0.68 [0.47–0.99], *p* = 0.041) and perceived stress (every 5 points increase: *HR* 1.31 [1.09–1.58], *p* = 0.005) could predict the cardiovascular outcomes. However, both predictive values would decrease when they were simultaneously adjusted. After adjusting for the physical clinical features, the associated of perceived stress on cardiac (*HR* 1.25 [1.04–1.51], *p* = 0.020) and composite clinical outcomes (*HR* 1.24 [1.05–1.47], *p* = 0.011) persisted.

**Conclusions:** For the patients with CHD, perceived stress strongly correlates with APTT. The activation of the intrinsic coagulation pathway is one of the mechanisms that high perceived stress causes cardiovascular events. This hints at an important role of the interaction of mental stress and coagulation function on cardiovascular prognosis. More attention needs to be paid to the patients with CHD with high perceived stress.

## Introduction

Although several large observational studies focusing general population have demonstrated that high perceived stress is associated with increased incidence of coronary heart disease (CHD) and risk for cardiovascular events (1–3), the underlying mechanisms of the negative influence of perceived stress on the cardiovascular system are rarely studied. Coagulation activation has undoubtedly played an important role in the pathogenesis of intracoronary thrombi, which further leads to the occurrence of cardiovascular events. However, although several possible mechanisms (altered automatic nervous system activity, elevated inflammation activity, etc.) have been reported to explain the linkage of psychological distress on the increased risk of cardiovascular events (4), the involvement of activated coagulation and fibrinolysis system has only been mentioned in a few articles (5, 6).

As is known, coagulation can be activated by the two fundamental approaches, the intrinsic pathway reflected by activated partial thromboplastin time (APTT), and the extrinsic pathway reflected by prothrombin time (PT), which converge to a common pathway of thrombosis. Evidence has emerged that in an animal experiment, shortened APTT, PT, and elevated fibrinogen could be induced by chronic localized cold stress (7). In human studies, the acute stressful laboratory tasks can cause a transient activation of coagulation and fibrinolysis in the general population and patients with major depression disorder (8–10). Besides, a shortened APTT more frequently occurred in patients with acute coronary syndromes (ACSs) (11). Outbursts of anger have been demonstrated to be related to the occurrence of acute myocardial infarction (AMI) and ischemic stroke (12). Research on long-term stress has found a heightened level of fibrinogen in subjects with job strain or mood disorders (8). One prior study observed a significant negative correlation between depression and APTT in the participants with low perceived social support (13). Therefore, it is reasonable to hypothesize that high perceived stress could through activating coagulation system to promote the occurrence of cardiovascular events. However, to the best of our knowledge, the studies concerning the relationships among the perceived stress, coagulation, and cardiac outcomes have previously not been published.

Thus, this study aimed to investigate the association between perceived stress and coagulation function, especially the intrinsic pathway coagulation function, and to explore the predictive effect of both on the cardiac and composite clinical outcomes.

## Methods

### Patient and Public Involvement

**Figure 1:**
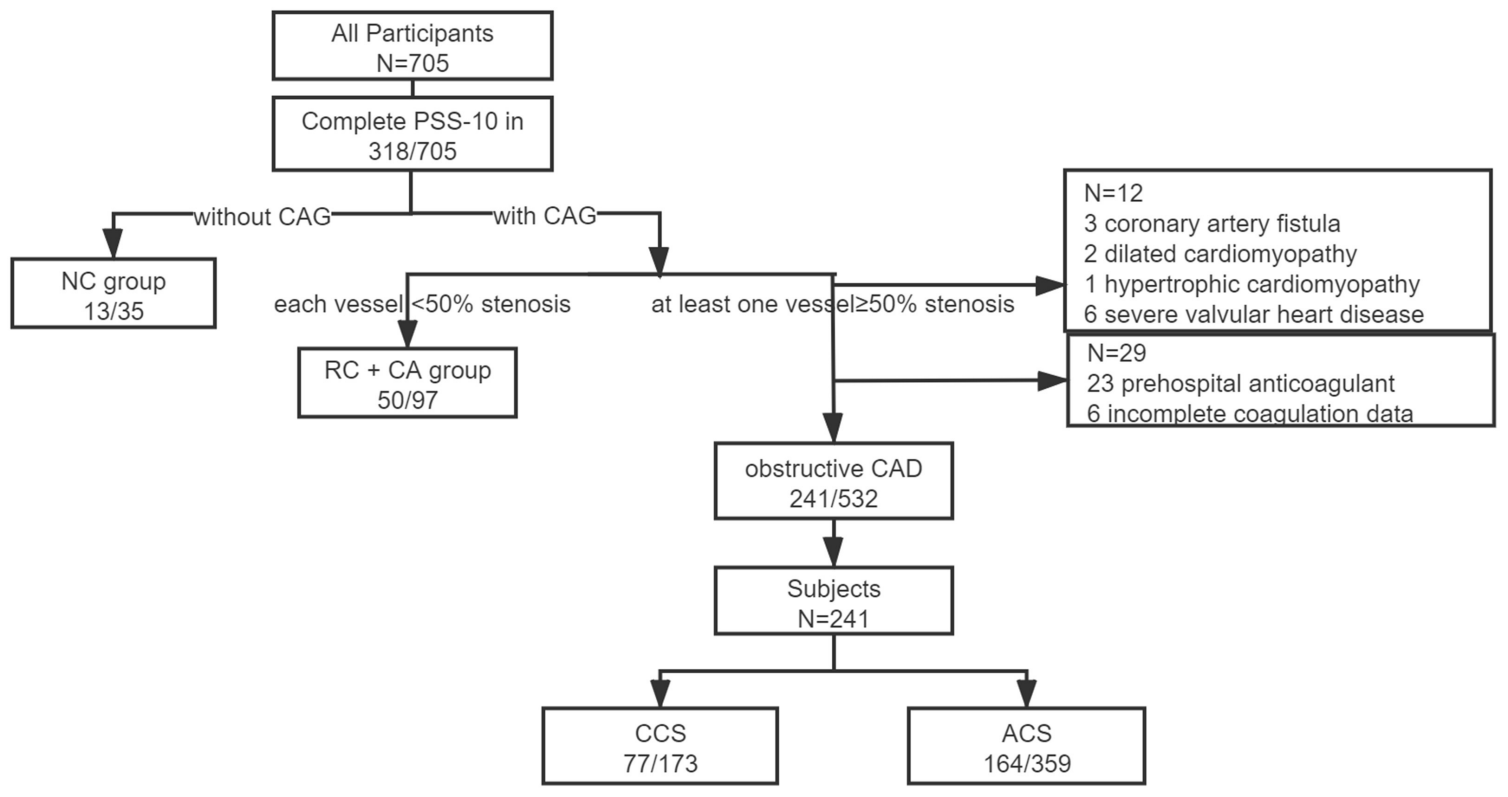
Patient recruitment. Obstructive coronary artery disease (CAD) indicated with at least one obstructive vessel (≥50%) presented in coronary angiography during this hospitalization or with a history of coronary artery bypass grafting or coronary stent implantation. NC, without coronary angiography; RC, rule out of CAD; CA, coronary atherosclerosis; CCS, chronic coronary syndrome; ACS, acute coronary syndrome; and CAD, coronary artery disease.

This is an exploratory analysis of a perspective cohort based on a cross-sectional study for investigating the psychological status of inpatients with admitted diagnose of CHD. The detailed protocol has been published (14). In brief, 705 consecutive inpatients with a main admitting diagnosis of CHD and without the urgency for emergency percutaneous coronary intervention (PCI) or intensive care, in the Guangdong Provincial People's Hospital were surveyed by the Patient Health Questionnaire (PHQ-9), Generalized Anxiety Questionnaire-7 Scale (GAD-7), and 10-item Perceived Stress Scale (PSS-10) between October 2017 and January 2018 under the supervision of a well-trained psycho-cardiologist on the day before coronary angiography (CAG). The patients were categorized based on the outcomes of CAG and discharge diagnosis, and further classified as a chronic coronary syndrome (CCS) or ACS according to the European Society of Cardiology (ESC) guidelines (15). Since PSS-10 was an optional item, 252 obstructive patients with CHD finally finished the questionnaire. We further excluded the patients with prehospital use of anticoagulants and incomplete data for coagulation function, leaving 241 subjects into the analyses (Figure 1). Follow-up data were collected at the 6th month and later yearly through a scripted telephone interview. The participants were queried for the occurrence of cardiac and non-cardiac rehospitalization, myocardial infarction, stroke, revascularization, and death. Major adverse cardiovascular event (MACE) was defined as a composite of cardiac revascularization, cardiovascular death, non-fatal AMI, non-fatal stroke, or urgent coronary revascularization. Composite endpoint was defined as any occurrence of events mentioned above. The patients were not involved in the recruitment and conduct of the study.

The study complied with the Declaration of Helsinki and was approved by the Medical Ethics Committee of Guangdong Provincial People's Hospital with the following reference number: No.GDREC2017203H. Clinical data were obtained from the medical records. All the participants gave written informed consent before inclusion.

### Coagulation Function Test

To avoid the interference of possible prescription of low molecular weight heparin (LMWH) to the patients with ACS, the samples of venous blood were drawn within 1 h after admission for coagulation function test and other biochemical analyses. A comparatively calm state of subjects was warranted before blood drawing and psychological assessment.

Activated partial thromboplastin time, PT, TT, fibrinogen, and D-dimer were assayed on the STA-R Evolution® System (Beijing Stago Diagnosis Trading Co., Ltd., Beijing, China) with the accompanying reagents of the instrument (Diagnostica Stago, Taverny, France). The reference ranges of APTT were 30.0–45.0 s given by the Clinical Laboratory and the mean value of APTT in our study was 37.95 s. A shortened APTT was defined as a specimen exhibiting an APTT of <35.0 s (lower quartile).

### Assessment of Perceived Stress

Perceived stress status was evaluated with the Chinese version of PSS-10 (as shown in Additional File 1), which as a short form of PSS-14 (16) has been validated in the Chinese cardiac patients (17). The patients were asked about how often in the last month, they felt (1) upset by something happening unexpectedly; (2) unable to control important things in life; (3) nervous and stressed; (4) confident about the ability to handle personal problems; (5) that things were going as expected; (6) unable to cope with all the things had to do; (7) able to deal with irritations; (8) on top of things; (9) angered because of things that happened out of control; and (10) unable to overcome piled up difficulties. Each item was scored on a 5-point scale ranging from 0 = never to 4 = very often. Higher score represented higher stress level. A well-acknowledged cutoff for PSS-10 did not exist, however, to differentiate, we defined the upper quartile as the threshold (PSS-10 > 16) for binary classification.

### Assessment of Depression and Anxiety

The Chinese version of PHQ-9 (18) and GAD-7 (19) were applied for evaluating the depression and anxiety symptoms of patients. Each questionnaire, rated on a 0–3 Likert-type scale with a higher score on each item representing more frequently being bothered by the symptom in the last 2 weeks, has demonstrated good validity and reliability among the Chinese cardiac patients (20, 21).

### Creatinine Clearance (CCR) and Coronary Artery Stenosis

Creatinine clearance (CCR) was estimated by using the Cockcroft-Gault formula with the value of serum creatinine tested at admission.

Coronary artery stenosis was assessed by CAG during this hospitalization. A 3-vessel obstructive coronary artery disease (CAD) referred to the presence of obstructive stenosis (≥50%) in CAG or history of coronary artery bypass grafting or stent implantation, in all the three main epicardial coronary arteries.

### Other Outcomes

The results of other laboratory examinations, echocardiography, and medical history were acquired from the medical records.

### Statistical Analysis

The clinical characteristics were reported as rate (percentages) for the categorical variables and mean ± SD or median (interquartile range [IQR]) for continuous variables. The homogeneity of clinical characteristics between those who completed and did not complete the questionnaire was first analyzed. The differences in baseline characteristics from the perspective of perceived stress status (PSS ≤ 16 or >16) and APTT (APTT ≤ 35.0 s or >35.0 s) were both compared with Student's *t*-test or Wilcoxon rank-sum test for comparing the continuous variables, and the chi-square test or Fisher's exact test for categorical variables as appropriate. The risk ratio of a high perceived stress status on shortened APTT was analyzed using a forward stepwise logistic regression model, such as covariates with age, sex, body mass index (BMI), CCR, level of low-density lipoprotein cholesterol (LDLC), PT, type of CHD, comorbid hypertension, and diabetes. Spearman's correlation was applied to describe the association between APTT and perceived stress, depression, or anxiety score. Multiple linear analyses for each APTT parameter with a PSS-10 score were analyzed, taking PT as another confounding variable after the selection using a forward stepwise method. To avoid the risk of prolonged APTT caused by unrecorded prior intake of coagulants, four cases with large-standardized residuals (>+3) were not included in multiple linear models. The Kaplan–Meier survival analyses were conducted to determine the influence of high perceived stress and shortened APTT status on the clinical outcomes. Cox regression analyses were used with APTT and PSS-10 score individually and simultaneously being taken into the models. In addition, we testify the stability of the outcomes through the stepwise addition of confounding factors. Statistical analysis was performed using SAS 9.4 software. A value of *p* < 0.05 was considered statistically significant.

## Results

### Clinical Characteristics

After ruling outpatients with incomplete data or with prehospital use of anticoagulants, 241 out of 532 inpatients with a mean age of 62.12 ± 10.47 years were included in the analyses. The median (IQR) score of PSS-10 was 11 (7–16) points. The prevalence of concurrent hypertension and concurrent diabetes were 63.5 and 34.4%. Additionally, 68.0% of these patients were diagnosed with ACS. During a median follow-up of 26 months, 49 major adverse cardiovascular events, 21 non-cardiac rehospitalization, and 67 cases of composite endpoint happened in 208 patients (follow-up rate 86.3%).

Compared to the ones who did not finish the questionnaire, those who completed PSS-10 were younger, with better renal function, lower LDL-C, and higher PHQ-9 and GAD-7 scores (as shown in Supplementary Table 1). Since PSS-10 was an optional item for the patients, the patients with less difficulties in reading and writing and more psychological disturbance might be more willing to complete the survey. However, no significant difference was found in coronary artery condition, ejection fraction, comorbid diabetes or hypertension, and coagulation function. The ratio of completers over the participants maintained around 45% regardless of the categorization of patients (CCS: 44.5%; ACS: 45.7%).

### Perceived Stress and APTT

The comparisons of the baseline characteristics between the groups categorized by PSS > 16 vs. PSS ≤ 16, and APTT ≤ 35.0 s vs. >35.0 s are presented in Tables 1, 2. The patients with a high perceived stress status had shorter APTT (36.71 vs. 38.45, *p* = 0.009), accompanied by elevated D-dimer level (*p* = 0.032) and depression (*p* < 0.001) and anxiety symptom scores (*p* < 0.001). No significant difference was found in the comparisons in PT, TT, fibrinogen, and the other clinical features, such as renal and hepatic function. Simultaneously, shortened APTT status was significantly associated with increased PSS-10 score (14.67 vs. 11.22, *p* = 0.003). Interestingly, a closer linkage of APTT with perceived stress rather than with depression (*p* = 0.012) or anxiety (*p* = 0.29) was observed. Furthermore, Spearman's correlation coefficient showed that the correlation of APTT with perceived stress score (*r*_s_ = −0.20, *p* = 0.002) was much stronger than with depression (*r*_s_ = −0.13, *p* = 0.054) or anxiety symptom score (*r*_s_ = −0.037, *p* = 0.57).

**Table 1:** Comparisons of the baseline characteristics between the groups categorized by perceived stress status.

	**Total**	**PSS > 16**	**PSS ≤ 16**	***p*-value**
	***n* = 241**	***n* = 59**	***n* = 182**	
Age (years)	62.12 (10.47)	62.05 (11.63)	62.14 (10.10)	0.95
Body mass index (kg/m^2^)	24.60 (2.95)	24.68 (3.36)	24.57 (2.81)	0.80
**Gender (male/female)**	190/51	41/18	149/33	**0.043**
CCS/ACS (No./No.)	77/164	14/45	63/119	0.12
Diabetes [No. (%)]	83 (34.44)	20 (33.90)	63 (34.62)	0.92
Hypertension [No. (%)]	153 (63.49)	42 (71.19)	111 (60.99)	0.16
3-vessel obstructive CAD [No. (%)]	146 (60.58)	33 (55.93)	113 (62.09)	0.40
Ejection Fraction (%)	58.38 (11.00)	58.20 (11.22) *N* = 56	58.45 (10.96) *N* = 166	0.88
Creatinine Clearance^a^ (ml/min)	68.79 (22.33)	68.64 (26.16)	68.84 (21.02)	0.96
Total Bilirubin^b^ (μmol/L)	13.08 (7.15)	12.78 (9.15)	13.18 (6.40)	0.13
ALT^b^ (U/L)	30.30 (27.82)	25.75 (16.10)	31.78 (30.58)	0.14
LDLC (mmol/L)	2.78 (0.83)	2.84 (0.80)	2.77 (0.84)	0.56
**APTT (s)**	38.02 (4.47)	36.71 (4.81)	38.45 (4.28)	**0.009**
**APTT** **≤** **35.0 (s)/ APTT** **>** **35.0 (s)** (No./No.)	58/183	23/36	35/147	**0.002**
PT (s)	13.57 (0.80)	13.53 (0.70)	13.58 (0.82)	0.64
TT (s)	16.73 (1.58)	16.82 (1.39)	16.70 (1.64)	0.62
Fibrinogen (g/L)	4.03 (1.16)	4.22 (1.38)	3.97 (1.07)	0.22
**D-dimer (μg/L)**	370 (270–610)	430 (310–780)	360 (270–540)	**0.032**
**Perceived stress score** ^b^	12.05 (6.43)	20.64 (4.66)	9.27 (4.00)	** <0.001**
**Depression symptom score** ^b^	5.11 (4.82)	8.63 (6.61)	3.97 (3.38)	** <0.001**
**Anxiety symptom score** ^b^	3.68 (4.26)	7.76 (5.51)	2.36 (2.68)	** <0.001**

*Note: Continuous variables are given as mean (SD) or median (interquartile range)*.

a *Creatinine clearance is estimated using the Cockcroft-Gault formula*.

b *Data is presented as mean (SD), however, the difference between the groups is calculated using Wilcoxon rank-sum test*.

*Variable marked in bold represents that statistically significant difference exists between groups*.

**Table 2:** Comparisons of the baseline characteristics between the groups categorized by activated partial thromboplastin time (APTT).

	**Total**	**APTT ≤ 35s**	**APTT > 35s**	***p*-value**
	***n* = 241**	***n* = 58**	***n* = 183**	
Age (years)	62.12 (10.47)	62.59 (12.51)	61.97 (9.77)	0.73
Body mass index (kg/m^2^)	24.60 (2.95)	24.38 (3.33)	24.66 (2.83)	0.52
Gender (male/female)	190/51	43/15	147/36	0.31
CCS/ACS (No./No.)	77/164	18/40	59/124	0.86
Diabetes [No. (%)]	83 (34.44)	23 (39.66)	60 (32.79)	0.34
Hypertension [No. (%)]	153 (63.49)	41 (70.69)	112 (61.20)	0.19
3-vessel obstructive CAD [No. (%)]	146 (60.58)	37 (63.79)	109 (59.56)	0.57
Ejection Fraction (%)	58.38 (11.00)	60.00 (11.22) *N* = 53	57.88 (10.91) *N* = 169	0.22
Creatinine Clearance^a^ (ml/min)	68.79 (22.33)	68.25 (25.21)	68.96 (21.41)	0.83
Total Bilirubin^b^ (μmol/L)	13.08 (7.15)	12.26 (8.82)	13.34 (6.54)	0.074
ALT^b^ (U/L)	30.30 (27.82)	25.76 (16.93)	31.74 (30.38)	0.070
LDLC (mmol/L)	2.78 (0.83)	2.93 (0.79)	2.74 (0.84)	0.13
**APTT (s)**	38.02 (4.47)	32.92 (1.79)	39.64 (3.79)	** <0.001**
**PT (s)**	13.57 (0.80)	13.30 (0.60)	13.66 (0.83)	**0.003**
TT (s)	16.73 (1.58)	16.61 (0.94)	16.77 (1.74)	0.36
Fibrinogen (g/L)	4.03 (1.16)	3.89 (0.91)	4.08 (1.22)	0.21
D-dimer (μg/L)	370 (270–610)	385 (270–645)	370 (270–610)	0.86
**Perceived stress score** ^b^	12.05 (6.43)	14.67 (7.69)	11.22 (5.75)	**0.003**
**PSS-10 score** **>16/≤16** (No./No.)	59/182	23/35	36/147	**0.002**
**Depression symptom score** ^b^	5.11 (4.82)	6.71 (6.11)	4.60 (4.23)	**0.012**
Anxiety symptom score^b^	3.68 (4.26)	4.52 (5.32)	3.42 (3.85)	0.29

*Note: Continuous variables are given as mean (SD) or median (interquartile range)*.

a *Creatinine clearance is estimated using the Cockcroft-Gault formula*.

b *Data is presented as mean (SD), however, the difference between the groups is calculated using Wilcoxon rank-sum test*.

*Variable marked in bold represents that statistically significant difference exists between groups*.

The multivariate analyses using a forward stepwise logistic regression model taking all the physical features into analysis revealed that PT and perceived stress status (odds ratio [*OR*] 2.75 (95% *CI* 1.42–5.33), *p* = 0.003) were the only two remaining factors that associated with shortened APTT. This association kept marginal significant [*OR* 2.05 (95% *CI* 0.98–4.30), *p* = 0.056] after adding PHQ-9 score into adjustment. Multiple linear regression models indicated that every single point increase in PSS-10 was relevant to approximately 0.13 s decrease in APTT, regardless of the type of coronary heart disease (for all *p* = 0.001: CCS: 0.16 s, *p* = 0.021; ACS: 0.12 s, *p* = 0.011) (Table 3).

**Table 3:** Multiple linear regression models of APTT in the patients with the chronic coronary syndrome (CCS) and acute coronary syndrome (ACS).

**Variables**	**Total**		**CCS**		**ACS**	
	**Regression coefficient (95% CI)**	***p*-value**	**Regression coefficient (95% CI)**	***p*-value**	**Regression coefficient (95% CI)**	***p*-value**
Intercept	28.46 (20.11, 36.81)	<0.001	24.13 (8.90,39.35)	0.002	30.55 (20.48, 40.62)	<0.001
PSS-10 score	−0.13 (−0.20, −0.05)	**0.001**	−0.16 (−0.29, −0.02)	**0.021**	−0.12 (−0.22, −0.03)	**0.011**
PT	0.80 (0.19, 1.40)	**0.011**	1.10 (−0.01, 2.21)	0.053	0.70 (−0.08, 1.39)	0.078

*Note: Multiple linear regression analyses treat APTT as the dependent variable, and 10-item Perceived Stress Scale (PSS-10) score, prothrombin time (PT), and low-density lipoprotein cholesterol (LDLC) as predictors*.

*Variable marked in bold represents that statistically significant difference exists between groups*.

### Perceived Stress, APTT, and Prognosis

From the perspective of clinical outcome (Table 4), the patients with MACE had significantly shorter APTT (*p* = 0.041), higher PSS-10 (*p* = 0.051), and PHQ-9 scores (*p* = 0.030). The occurrence of composite endpoint was associated with PSS-10 (*p* = 0.021) and depression symptom scores (*p* = 0.017). Non-cardiac rehospitalization was irrelevant to both perceived stress and APTT. Similarly, the Kaplan–Meier survival analyses (as shown in Supplementary Figures 1, 2) exhibited an obvious tendency of a negative influence of high perceived stress status on MACE (*p* = 0.095) and composite outcomes (*p* = 0.049).

**Table 4:** Comparison of APTT, PSS-10, Patient Health Questionnaire (PHQ-9), Generalized Anxiety Questionnaire-7 Scale (GAD-7) scores for different clinical outcomes.

**Variables**	**MACE**			**Non-cardiac rehospitalization**			**Composite endpoint**		
	**Happened**	**Not happened**	***p*-value**	**Happened**	**Not happened**	***p*-value**	**Happened**	**Not happened**	***p*-value**
APTT (s)	36.68 (3.81)	37.98 (3.88)	**0.041**	37.73 (3.51)	37.77 (3.93)	0.97	37.24 (3.98)	37.87 (3.84)	0.28
PSS-10 score^a^	14.24 (8.41)	11.18 (5.40)	**0.051**	12.86 (5.32)	11.77 (6.30)	0.18	13.78 (7.77)	11.01 (5.36)	**0.021**
PHQ-9 score^a^	6.78 (6.31)	4.51 (3.90)	**0.030**	6.24 (4.17)	4.76 (4.40)	0.064	6.48 (5.88)	4.36 (3.79)	**0.017**
GAD-7 score^a^	4.67 (5.76)	3.30 (3.44)	0.53	4.19 (4.11)	3.51 (3.97)	0.35	4.55 (5.41)	3.18 (3.29)	0.33

a *Data are presented as mean (SD), however, the difference between the groups is calculated using Wilcoxon rank-sum test*.

*Variable marked in bold represents that statistically significant difference exists between groups*.

We tried taking APTT and PSS-10 scores individually or simultaneously into the different Cox proportional hazard models (Table 5) and found that in the univariate analyses both APTT (every 5 s increase: hazard ratio [*HR*] 0.68 (95% *CI* 0.47–0.99), *p* = 0.041) and PSS-10 score (every 5 points increase: *HR* 1.31 (95% *CI* 1.09–1.58), *p* = 0.005) could predict the cardiovascular prognosis. Only PSS-10 score was associated with composite outcomes [every 5 points increase: *HR* 1.26 (95% *CI* 1.07–1.48), *p* = 0.006]. Different adjustments for other clinical variables proved that the statistical results were stable. Although the significance of the PSS-10 score was retained, the predictive values of APTT were to some extend decreased. Both the correlations of APTT and PSS-10 score with the clinical outcomes weakened when they were simultaneously incorporated into the models. After taking APTT and other physical characteristics into adjustment, PSS-10 score was still associated with increased risk for MACE [every 5 points increase: *HR* 1.25 (95% *CI* 1.04–1.51), *p* = 0.020] and composite endpoint [every 5 points increase: *HR* 1.24 (95% *CI* 1.05–1.47), *p* = 0.011], however, the statistical significance of APTT for MACE disappeared.

**Table 5:** Comparison of hazard ratios (*HR*s) of APTT, PSS-10, the score for a major adverse cardiovascular event (MACE), and composite endpoint among the different Cox proportional hazard models.

**Variables**	**MACE**				**Composite endpoint**			
	**APTT (s)**		**PSS-10 score**		**APTT (s)**		**PSS-10 score**	
	**HR^a^ (95% CI)**	***p*-value**	**HR^b^ (95% CI)**	***p*-value**	**HR^a^ (95% CI)**	***p*-value**	**HR^b^ (95% CI)**	***p*-value**
model 1a	0.68 (0.47–0.99)	**0.041**			0.82 (0.60–1.12)	0.21		
model 1b			1.31 (1.09–1.58)	**0.005**			1.26 (1.07–1.48)	**0.006**
model 1c	0.75 (0.52–1.08)	0.12	1.27 (1.05–1.53)	**0.016**	0.88 (0.65–1.19)	0.40	1.24 (1.05–1.47)	**0.010**
model 2a	0.71 (0.49–1.03)	0.070			0.84 (0.61–1.15)	0.28		
model 2b			1.28 (1.06–1.54)	**0.011**			1.25 (1.06–1.48)	**0.008**
model 2c	0.76 (0.53–1.08)	0.13	1.25 (1.03–1.51)	**0.022**	0.89 (0.65–1.21)	0.45	1.24 (1.05–1.47)	**0.013**
model 3a	0.71 (0.49–1.04)	0.071			0.83 (0.61–1.14)	0.26		
model 3b			1.28 (1.06–1.54)	**0.010**			1.26 (1.06–1.48)	**0.007**
model 3c	0.76 (0.53–1.09)	0.13	1.25 (1.04–1.51)	**0.020**	0.88 (0.64–1.19)	0.40	1.24 (1.05–1.47)	**0.011**

*model 1a clinical outcome = APTT; model 1b clinical outcome = PSS-10; model 1c clinical outcome = APTT + PSS-10*.

*model 2a clinical outcome = model 1a + age+ sex + CCR + CHD categorization^c^; model 2b clinical outcome = model 1b + age+ sex + CCR + CHD categorization^c^*.

*model 2c clinical outcome = model 1c + age+ sex + CCR + CHD categorization^c^; model 3a clinical outcome = model 2a + BMI + LDLC + hypertension + diabetes*.

*model 3b clinical outcome = model 2b + BMI+ LDLC + hypertension + diabetes; model 3c clinical outcome = model 2c + BMI + LDLC + hypertension + diabetes*.

a *Hazard Ratio for every 5 s increase*;

b *HR for every 5 points increase*;

*^c^categorization of chronic coronary syndrome or acute coronary syndrome*.

*Variable marked in bold represents that statistically significant difference exists between groups*.

## Discussion

Our research through both the univariate and multivariate analyses demonstrated that high perceived stress was associated with shortened APTT in patients with CHD. The classification of CCS or ACS did not affect the establishment of the conclusion. As compared with depression or anxiety, perceived stress exhibited a closer linkage to APTT. Shortened APTT and increased perceived stress were both individually related to worse cardiovascular prognosis, however, the effect of APTT on MACE could be diluted when adjusting for perceived stress. This hinted that the negative impact of perceived stress on cardiovascular outcomes might act partially through the mechanism of a hypercoagulable state in the intrinsic pathway. After adjusting for all clinical features, high perceived stress remained significantly associated with MACE and composite outcomes.

Though contradicted by few articles (13), APTT was reported irrelevant with depression or anxiety (5, 22), which was demonstrated by our research. Considering the strong correlation between mood disturbance and perceived stress, it was interesting to reveal an intensive linkage between APTT and perceived stress. As compared with mood disturbance, perceived stress measured by PSS-10 could be a more accurate indicator representing the stress received by the human body. Although to the best of our knowledge, the linkage between APTT and perceived stress has not been reported before, the findings that shortened APTT and platelet hyperreactivity in the patients with mood disorders would be reversed after the improvement of psychiatric symptoms (23, 24) might confirm the rationality.

The mechanisms that underlie the association between perceived stress and APTT are still poorly understood. Previous research toward the Chinese population reported a linkage between the PSS-14 score and the level of epinephrine and norepinephrine (25). It is hypothesized that catecholamine and cortisol under the control of perceived stress may exert an important influence on hemostasis (26–28). Another possible explanation is the elevation of intrinsic coagulation factors. It was revealed by Doulalas et al. (29) that more coagulation factors VII and X were expressed in the healthy individuals with depressed moods. Similarly, elevated factors VII and VIII were observed closely correlated with job strain among Korean male workers (30).

In accordance with previous literature (31–33), our results demonstrated that a hypercoagulability state predicted a worse cardiovascular prognosis. Although perceived stress has been linked to the increased cardiovascular risk in the general population for a long time (1–3), the research on the influence of perceived stress aiming at the patients with CHD has rarely been reported. Our study revealed that elevated perceived stress was associated with the increased risk of MACE and adverse composite outcomes, which was in line with a previous article reporting that a moderate or high perceived stress status at the time of AMI predicted adverse long-term outcomes (34). As indicated by the results, the negative influence of shortened APTT and elevated perceived stress on cardiovascular prognosis are to some extend overlapped, which means that shortened APTT might be one of the mechanisms that high perceived stress causes cardiovascular events.

As mentioned before, both acute and chronic mental stress can cause a gentle activation of coagulation (35) as well as increases in some coagulation factors, for example, FVIII. Meanwhile, shortened APTT analyzed in this study is mostly within a physiological range. How the minor change leads to a worse cardiovascular prognosis? Emerging data have demonstrated that mental stress only exerted weak effects on the development of CHD in healthy individuals (36), whereas it might play a critical role for a more severe progression and clinical outcome in the patients with a previous cardiovascular disease (34, 37). In our study, the pathological basis of CHD may have contributed to the vulnerability. Recently, a high FVIII level was found causally related to both CHD and venous thrombosis risk (38). Although vulnerable plaques are regarded as the culprit factor for a worse cardiac prognosis, “blood prone to thrombosis” has played an important role (31). Despite the overactivation of the sympathetic nerve system under stress, which might cause the mismatch of oxygen demand and supply for myocardium, mental stress-induced prolonged platelet activation (39, 40) and endothelial dysfunction (41) could be another precipitating factor. With the increased susceptibility of the endothelium to injury, endothelium lesions caused by ischemia or the rupture of plaque, through exposing procoagulant materials to the blood, might catalyze the initialization of thrombosis, and could be further strengthened under the push of high perceived stress through a hypercoagulable state and platelet activation. The alterations in inflammation may be another possible involved mechanism since it can be induced by mental stress (42), coupled with the activation of coagulation function, and is associated with an increased risk of cardiovascular diseases (43).

These findings point out the important role that the interaction of mental stress and coagulation function has played in the occurrence of cardiovascular events in patients with CHD and call for attention to the psychological state of patients in clinical practice. More attention needs to be paid to high perceived stress status due to the close linkage with biological measurements and worse prognosis. In addition, it is worthwhile to explore the benefits of stress management for patients with CHD. Several randomized clinical trials have already proved the effectiveness for improving clinical prognosis (44).

Additionally, mounting evidence has suggested that anticoagulant plays a crucial part in the therapy for ACS especially in the acute phase (45). The combination of low dose anticoagulant plus aspirin can reduce the risk for cardiac events in patients with stable CHD (46), however, increase the possibility of major bleeding events. Whether perceived stress could become an important indicator guiding the anticoagulant strategy needs to be further studied.

Our findings should be considered in light of several potential limitations. First, this is a single center observational study with a limited sample size. Second, the markers for coagulation factors and fibrinolysis and platelet activity were not tested in this study. Fortunately, the values of D-dimer and fibrinogen could at least provide some insights into the changes. Third, smoking could influence coagulation, fibrinolysis system, and platelet activation to promote the thrombi formation (47), but the smoking status was not included in our analyses due to the inaccurate data from medical records. However, based on the inaccurate data, we have roughly estimated the ratio of current smokers between APTT ≤ 35.0 s and >35.0 s, the difference was not significant. Fourth, since the PSS-10 was given to the patients as an optional item to avoid subjects filling out the questionnaire blindly, whether the completers could represent the whole CHD population needs to be explored. However, through the comparisons, a huge difference in physical features was not found.

## Conclusions

Perceived stress strongly correlates with APTT regardless of the type of CHD. The negative impact of high perceived stress on cardiovascular prognosis could partially be explained by the activation of the intrinsic coagulation pathway. These findings highlight the importance to recognize high perceived stress status in patients with CHD and indicate a vital role of the interaction between mental stress and coagulation function for cardiovascular events. Further research is needed to explore the deeper mechanisms underlying the phenomenon.

## Data Availability Statement

The raw data supporting the conclusions of this article will be made available by the authors, without unduereservation.

## Ethics Statement

The studies involving human participants were reviewed and approved by Medical Ethics Committee of Guangdong Provincial People's Hospital. The patients/participants provided their written informed consent to participate in this study.

## Author Contributions

HY surveyed all the patients at baseline. YL, AL, HW, and FL followed all patients. HY and XC collected and entered data into the database. XC, YL, and AL did the statistical analyses. HY and XC wrote the manuscript. QG, HM, and LG were senior physicians principally responsible for the study. HM and QG revised the manuscript. All the authors read and approved the final manuscript.

## Funding

This study was supported by the grants of Natural Science Foundation of Guangdong Province (2019A1515011224), Guangdong Medical Science and Technology Research Fund (2019118152336191), Guangdong Provincial Bureau of Traditional Chinese Medicine (20201008), and the High-level Hospital Construction Project of Guangdong Provincial People's Hospital (DFJH201811, DFJH201922, and DFJH2020003).

## Conflict of Interest

The authors declare that the research was conducted in the absence of any commercial or financial relationships that could be construed as a potential conflict of interest.

## Publisher's Note

All claims expressed in this article are solely those of the authors and do not necessarily represent those of their affiliated organizations, or those of the publisher, the editors and the reviewers. Any product that may be evaluated in this article, or claim that may be made by its manufacturer, is not guaranteed or endorsed by the publisher.
