# Supplementary material for: High Perceived Stress May Shorten Activated Partial Thromboplastin Time and Lead to Worse Clinical Outcomes in Patients With Coronary Heart Disease
Source: Front Cardiovasc Med. 2021 Nov 29;8:769857. doi: 10.3389/fcvm.2021.769857 (PMC8667268; doi:10.3389/fcvm.2021.769857)
Supplement: Supplementary file 1 [file Data_Sheet_1.docx]

Supplementary Material

# Supplementary Table 1

| Sup Table 1. Comparision of clinical characteristics between patients with PSS-10 completed and PSS-10 uncompleted. | | | | |
| --- | --- | --- | --- | --- |
|  | Patients | PSS uncompleted | PSS completed | p-value |
|  | N=532 | N=291 | N=241 |  |
| **Age (years)** | 63.60(10.14) | 64.83(9.71) | 62.12(10.47) | **.002** |
| Body mass index (kg/m2) | 24.45(3.06) | 24.33(3.15) | 24.60(2.95) | 0.33 |
| Gender (male/female ) | 410/122 | 220/71 | 190/51 | 0.38 |
| CCS/ACS (No./No.) | 173/359 | 96/195 | 77/164 | 0.80 |
| Diabetes (No.(%)) | 186(34.96) | 103(35.40) | 83(34.44) | 0.82 |
| Hypertension (No.(%)) | 327(61.47) | 174(59.79) | 153(63.49) | 0.38 |
| CHD with 3 vessel lesions (No.(%)) | 328(61.65) | 182(62.54) | 146(60.58) | 0.64 |
| Ejection Fraction (%) | 58.19(10.92) | 58.01(10.87) N=251 | 58.38(11.00) N=222 | 0.71 |
| Creatinine Clearancea (ml/min) | 65.37(21.80) | 62.54(20.97) | 68.79(22.33) | **<.001** |
| **LDLC (mmol/L)** | 2.88(0.90) | 2.96(0.95) | 2.77(0.84) | **.021** |
| APTT (s) | 37.95(4.20) | 37.89(3.98) | 38.02(4.47) | 0.71 |
| APTT≤35.0(s)/ APTT>35.0(s) (No./No.) | 127/405 | 69/222 | 58/183 | 0.92 |
| PT (s) | 13.59(0.77) | 13.61(0.74) | 13.57(0.80) | 0.55 |
| TT (s) | 16.76(1.69) | 16.79(1.77) | 16.73(1.58) | 0.70 |
| Fibrinogen (g/L) | 4.07(1.14) | 4.10(1.13) | 4.03(1.16) | 0.49 |
| Depression symptom scoreb | 4.30(4.31) | 3.64(3.72) | 5.11(4.82) | **<.001** |
| Anxiety symptom scoreb | 3.18(3.73) | 2.76(3.17) | 3.68(4.26) | **.024** |
| Note: Continuous variables are given as mean±SD. | | | | |
| ^a^: Creatinine clearance is estimated using the Cockcroft-Gault formula. | | | | |
| ^b^: data is presented as mean±SD, however difference between groups is calculated using Wilcoxon rank-sum test. | | | | |

# Supplementary Table 2 (Detailed Table 1)

| **Sup Table 2 Comparisons of baseline characteristics between groups categorized by perceived stress status.** | | | | |
| --- | --- | --- | --- | --- |
|  | Total | PSS>16 | PSS≤16 | p-value |
|  | n=241 | n=59 | n=182 |  |
| Age (years) | 62.12(10.47) | 62.05(11.63) | 62.14(10.10) | 0.95 |
| Body mass index (kg/m^2^) | 24.60(2.95) | 24.68(3.36) | 24.57(2.81) | 0.80 |
| **Gender (male/female)** | 190/51 | 41/18 | 149/33 | **.043** |
| CCS/ACS (No./No.) | 77/164 | 14/45 | 63/119 | 0.12 |
| Diabetes (No.(%)) | 83(34.44) | 20(33.90) | 63(34.62) | 0.92 |
| Hypertension (No.(%)) | 153(63.49) | 42(71.19) | 111(60.99) | 0.16 |
| 3-vessel obstructive CAD (No.(%)) | 146(60.58) | 33(55.93) | 113(62.09) | 0.40 |
| Ejection Fraction (%) | 58.38(11.00) | 58.20(11.22) N=56 | 58.45(10.96) N=166 | 0.88 |
| Creatinine (μmol/L) | 95.98(25.41) | 96.49(32.94) | 95.81(22.54) | 0.88 |
| Creatinine Clearance^a^ (ml/min) | 68.79(22.33) | 68.64(26.16) | 68.84(21.02) | 0.96 |
| Total Bilirubin**^b^** (μmol/L) | 13.08(7.15) | 12.78(9.15) | 13.18(6.40) | 0.13 |
| Direct Bilirubin**^b^** (μmol/L) | 2.76(3.06) | 2.81(4.15) | 2.74(2.63) | .075 |
| ALT**^b^** (U/L) | 30.30(27.82) | 25.75(16.10) | 31.78(30.58) | 0.14 |
| GGT**^b^** (U/L) | 46.91(46.56) | 43.20(56.21) | 48.12(43.04) | 0.17 |
| ALP**^b^** (U/L) | 68.53(22.62) | 67.41(21.29) | 68.90(23.09) | 0.71 |
| Cholinesterase (U/L) | 7771(1469) | 7562(1587) | 7840(1427) | 0.21 |
| LDLC (mmol/L) | 2.78(0.83) | 2.84(0.80) | 2.77(0.84) | 0.56 |
| **APTT (s)** | 38.02(4.47) | 36.71(4.81) | 38.45(4.28) | **.009** |
| **APTT≤35.0(s)/ APTT>35.0(s)** (No./No.) | 58/183 | 23/36 | 35/147 | **.002** |
| PT (s) | 13.57(0.80) | 13.53(0.70) | 13.58(0.82) | 0.64 |
| TT (s) | 16.73(1.58) | 16.82(1.39) | 16.70(1.64) | 0.62 |
| Fibrinogen (g/L) | 4.03(1.16) | 4.22(1.38) | 3.97(1.07) | 0.22 |
| D-dimer (μg/L) | 370(270-610) | 430(310-780) | 360(270-540) | **.032** |
| **Perceived stress score^b^** | 12.05(6.43) | 20.64(4.66) | 9.27(4.00) | **<.001** |
| **Depression symptom score^b^** | 5.11(4.82) | 8.63(6.61) | 3.97(3.38) | **<.001** |
| **Anxiety symptom score^b^** | 3.68(4.26) | 7.76(5.51) | 2.36(2.68) | **<.001** |
| Note: Continuous variables are given as mean(SD) or median(interquartile range). | | | | |
| ^a^: Creatinine clearance is estimated using the Cockcroft-Gault formula. | | | | |
| ^b^: data is presented as mean(SD), however difference between groups is calculated using Wilcoxon rank-sum test. | | | | |

# Supplementary Table 3 (Detailed Table 2)

| **Sup Table 3 Comparisons of baseline characteristics between groups categorized by APTT.** | | | | |
| --- | --- | --- | --- | --- |
|  | Total | APTT≤35s | APTT>35s | p-value |
|  | n=241 | n=58 | n=183 |  |
| Age (years) | 62.12(10.47) | 62.59(12.51) | 61.97(9.77) | 0.73 |
| Body mass index (kg/m^2^) | 24.60(2.95) | 24.38(3.33) | 24.66(2.83) | 0.52 |
| Gender (male/female) | 190/51 | 43/15 | 147/36 | 0.31 |
| CCS/ACS (No./No.) | 77/164 | 18/40 | 59/124 | 0.86 |
| Diabetes (No.(%)) | 83(34.44) | 23(39.66) | 60(32.79) | 0.34 |
| Hypertension (No.(%)) | 153(63.49) | 41(70.69) | 112(61.20) | 0.19 |
| 3-vessel obstructive CAD (No.(%)) | 146(60.58) | 37(63.79) | 109(59.56) | 0.57 |
| Ejection Fraction (%) | 58.38(11.00) | 60.00(11.22) N=53 | 57.88(10.91) N=169 | 0.22 |
| Creatinine (μmol/L) | 95.98(25.41) | 94,58(28.53) | 96.42(24.40) | 0.63 |
| Creatinine Clearance^a^ (ml/min) | 68.79(22.33) | 68.25(25.21) | 68.96(21.41) | 0.83 |
| Total Bilirubin**^b^** (μmol/L) | 13.08(7.15) | 12.26(8.82) | 13.34(6.54) | .074 |
| Direct Bilirubin**^b^** (μmol/L) | 2.76(3.06) | 2.85(4.29) | 2.73(2.57) | 0.14 |
| ALT**^b^** (U/L) | 30.30(27.82) | 25.76(16.93) | 31.74(30.38) | .070 |
| GGT**^b^** (U/L) | 46.91(46.56) | 52.60(64.02) | 45.09(39.43) | 0.89 |
| ALP**^b^** (U/L) | 68.53(22.62) | 71.07(23.43) | 67.72(22.36) | 0.31 |
| Cholinesterase (U/L) | 7771(1469) | 8038(1571) | 7686(1429) | 0.11 |
| LDLC (mmol/L) | 2.78(0.83) | 2.93(0.79) | 2.74(0.84) | 0.13 |
| **APTT (s)** | 38.02(4.47) | 32.92(1.79) | 39.64(3.79) | **<.001** |
| **PT (s)** | 13.57(0.80) | 13.30(0.60) | 13.66(0.83) | **.003** |
| TT (s) | 16.73(1.58) | 16.61(0.94) | 16.77(1.74) | 0.36 |
| Fibrinogen (g/L) | 4.03(1.16) | 3.89(0.91) | 4.08(1.22) | 0.21 |
| D-dimer (μg/L) | 370(270-610) | 385(270-645) | 370(270-610) | 0.86 |
| **Perceived stress score^b^** | 12.05(6.43) | 14.67(7.69) | 11.22(5.75) | **.003** |
| **PSS-10 score >16 / ≤16** (No./No.) | 59/182 | 23/35 | 36/147 | **.002** |
| **Depression symptom score^b^** | 5.11(4.82) | 6.71(6.11) | 4.60(4.23) | **.012** |
| Anxiety symptom score^b^ | 3.68(4.26) | 4.52(5.32) | 3.42(3.85) | 0.29 |
| Note: Continuous variables are given as mean(SD) or median(interquartile range). | | | | |
| ^a^: Creatinine clearance is estimated using the Cockcroft-Gault formula. | | | | |
| ^b^: data is presented as mean(SD), however difference between groups is calculated using Wilcoxon rank-sum test. | | | | |

# Supplementary Figure 1

**
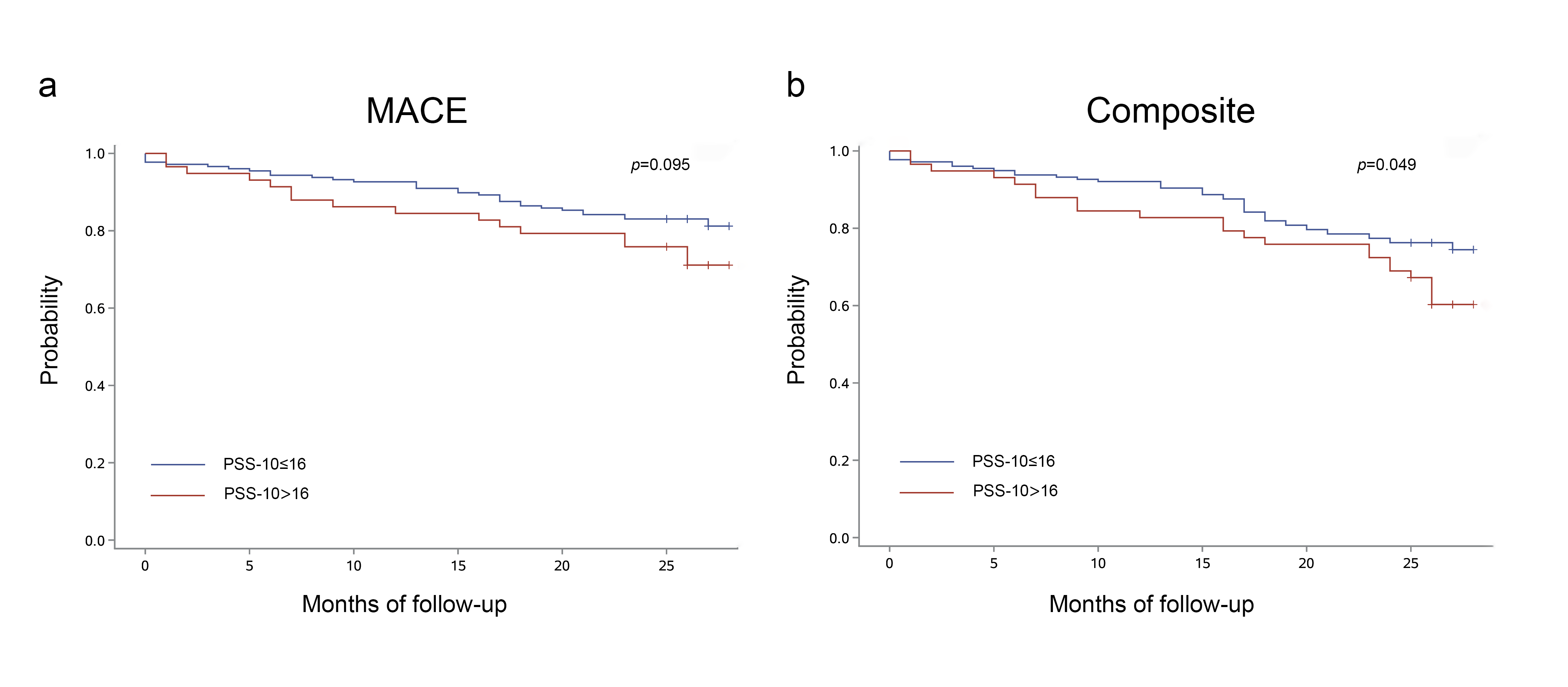
**

**Sup Figure 1**  **Kaplan-Meier plots for different perceived stress status (PSS-10≤16/ PSS-10>16) on (a) major adverse cardiovascular events and (b) composite outcomes.**

# Supplementary Figure 2

**
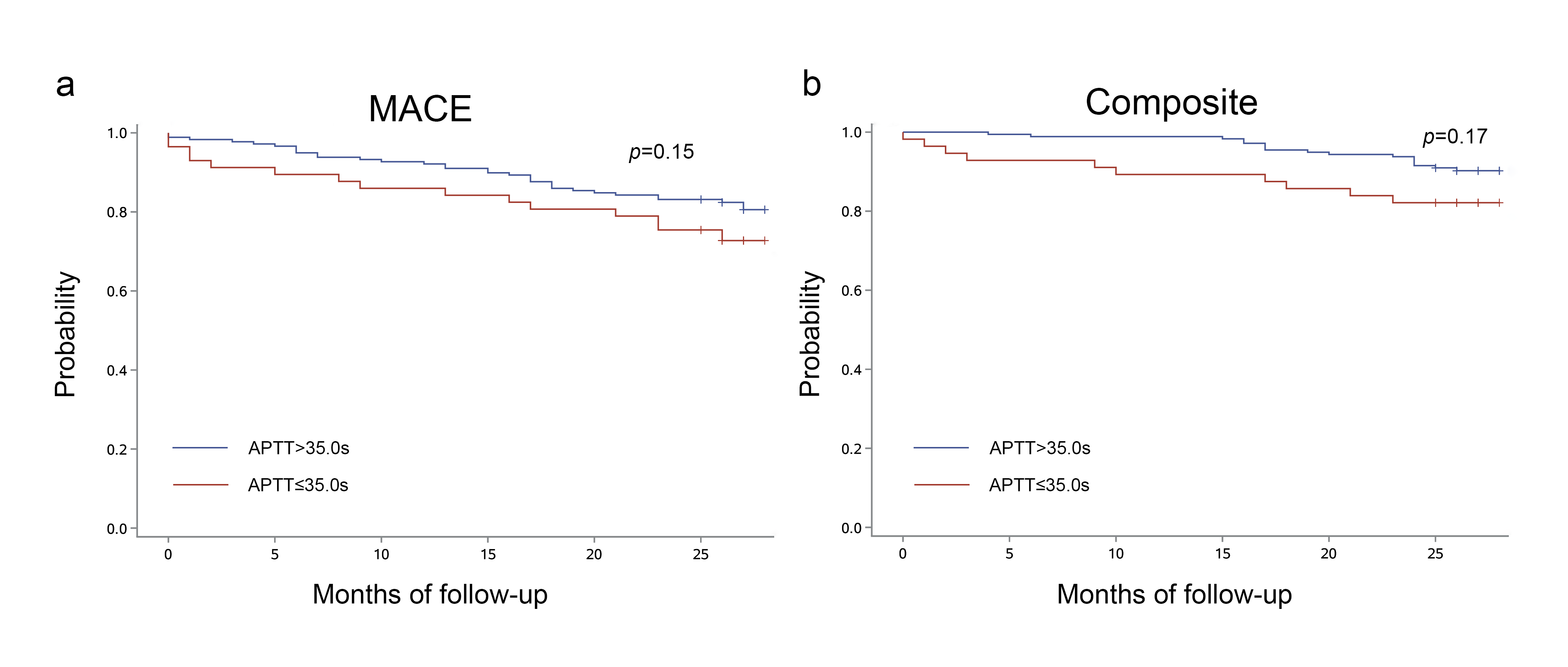
**

**Sup Figure 2**  **Kaplan-Meier plots for different APTT status (APTT≤35.0/ PSS-10>35.0) on (a) major adverse cardiovascular events and (b) composite outcomes.**
